# Feasibility and validity of using WHO adolescent job aid algorithms by health workers for reproductive morbidities among adolescent girls in rural North India

**DOI:** 10.1186/s12913-015-1067-x

**Published:** 2015-09-21

**Authors:** Siddaiah Archana, B. Nongkrynh, K. Anand, CS Pandav

**Affiliations:** Centre for Community Medicine, All India Institute of Medical Sciences, #14, Ground floor, Near old OT block, Ansari Nagar, New Delhi 110029 India

**Keywords:** Adolescent girls, Reproductive health, Female health workers, WHO adolescent Job Aid, India

## Abstract

**Background:**

High prevalence of reproductive morbidities is seen among adolescents in India. Health workers play an important role in providing health services in the community, including the adolescent reproductive health services. A study was done to assess the feasibility of training female health workers (FHWs) in the classification and management of selected adolescent girls’ reproductive health problems according to modified WHO algorithms.

**Methods:**

The study was conducted between Jan-Sept 2011 in Northern India. Thirteen FHWs were trained regarding adolescent girls’ reproductive health as per WHO Adolescent Job-Aid booklet. A pre and post-test assessment of the knowledge of the FHWs was carried out. All FHWs were given five modified WHO algorithms to classify and manage common reproductive morbidities among adolescent girls. All the FHWs applied the algorithms on at least ten adolescent girls at their respective sub-centres. Simultaneously, a medical doctor independently applied the same algorithms in all girls. Classification of the condition was followed by relevant management and advice provided in the algorithm. Focus group discussion with the FHWs was carried out to receive their feedback.

**Results:**

After training the median score of the FHWs increased from 19.2 to 25.2 (*p* – 0.0071). Out of 144 girls examined by the FHWs 108 were classified as true positives and 30 as true negatives and agreement as measured by kappa was 0.7 (0.5–0.9). Sensitivity, specificity, positive predictive value (PPV) and negative predictive value (NPV) were 94.3 % (88.2–97.4), 78.9 % (63.6–88.9), 92.5 % (86.0–96.2), and 83.3 % (68.1–92.1) respectively.

**Discussion:**

A consistent and significant difference between pre and post training knowledge scores of the FHWs were observed and hence it was possible to use the modified Job Aid algorithms with ease. Limitation of this study was the munber of FHWs trained was small. Issues such as time management during routine work, timing of training, overhead cost of training etc were not taken into account.

**Conclusions:**

Training was successful in increasing the knowledge of the FHWs about adolescent girls’ reproductive health issues. The FHWs were able to satisfactorily classify the common adolescent girls’ problems using the modified WHO algorithms.

**Electronic supplementary material:**

The online version of this article (doi:10.1186/s12913-015-1067-x) contains supplementary material, which is available to authorized users.

## Background

There are more than 600 million adolescent girls (10–19 years) in the developing world. Adolescents constitute 18–25 % of the population in South-east Asia region. In India, in 2011, the adolescent population constituted around 20.9 % of the total population [1]. Millions of adolescent girls living in developing countries remain invisible in national policies and programmes and belong to the vulnerable section of the society. Several studies in India have shown that the overall prevalence of reproductive morbidities and infections of the reproductive tract vary from 21 to 66 % [2–4]. Additionally, about 50 % of new HIV infections currently occur in people under 25 [5]. There are many factors which determine the health seeking behaviour of adolescent girls, which include traditional beliefs, myths and misconceptions.

Adolescents face many barriers in obtaining the health services. These barriers relate to the availability, accessibility, acceptability and equity of health services [6]. Health workers play an important role in providing health services in the community, and are in a good position to address issues related to the reproductive health of adolescents [7]. Promising benefits has been shown by interventions utilizing lay health workers for immunization, infectious diseases like tuberculosis, HIV, maternal, neonatal health care, screening, and diabetes management [8–12]. Training health staff to provide more youth-friendly health services can increase the utilization of reproductive health services for problems like STIs, especially among the youth [13].

The World Health Organization’s (WHO) focus is on making the adolescent health delivery accessible, acceptable, equitable, appropriate, and effective. Adolescent Reproductive and Sexual Health (ARSH) is a strategy which aims to address the factors that contribute to positive sexual and reproductive health outcomes. As part of ARSH strategy, the Adolescent Job Aid was developed by the WHO [14]. The Adolescent Job Aid takes into account that in most settings health workers will be providing primary health services to adolescents. It is a handy desk reference intended for health workers who provide primary care services to adolescents. It provides precise, step-by-step guidance through the use of simple algorithms on how to deal with adolescents when they present with a problem or a concern about their health or development. It has three parts viz clinical interaction between the adolescent and the health worker; application of algorithms by health worker for managing common adolescent conditions; information for adolescents and their parents on important health and development issues given by health worker. The main objective is to help health workers to respond to their adolescent patients more effectively. Considering the importance of addressing adolescent health issues, we attempted to adapt and test the Adolescent Job Aid in a pilot study among adolescent girls in a rural setting. This study was carried out to assess the feasibility of training female health workers (FHWs) in providing reproductive health services according to algorithms provided in the Adolescent Job Aid. This article discusses the process and the issues related to providing adolescent health services via health workers.

## Methods

### Study setting

The study was conducted between Jan-Sept 2011 in Comprehensive Rural Health Services Project (CRHSP), Ballabgarh, situated in the state of Haryana in Northern India. CRHSP includes 28 villages with a population of 90,240 in the year 2011 and adolescents constitute around 19 % of the total population, as per the routine demographic surveillance. CRHSP provides health care according to the national norms through two primary health centers, with six sub-centers each. Each of these sub-centers has one FHW, who is responsible for the reproductive health services in the sub-centre. The FHWs working in all the 12 subcentres were included in this study. These FHWs are auxiliary nurse midwives having basic nursing skills. They assist in the provision of maternal and newborn health care, particularly during the prenatal, natal, and postnatal periods.

### Training

A training manual was prepared in Hindi, for which main contents were taken from the WHO Adolescent Job Aid and text book of Gynaecology [14, 15] (Table 1). The manual was pretested with health workers from the urban field practice area and was finalized. All the FHWs were given one day training in their local language (Hindi) regarding adolescent girls’ reproductive health. The training was conducted using training guide, health education videos, interactive case discussion, and flip charts. As the videos used in the training were in English, videos were muted while playing, allowing the investigator to explain them in Hindi.

**Table 1:** Contents of training (adopted from Job Aid)

1. Introduction to adolescent issues
2. Female Reproductive System
3. Building Rapport with an Adolescent Girl
4. Delayed puberty
5. “I have a lot of pain during periods”
6. “I bleed a lot during my periods”
7. “I have irregular periods”
8. “I have an abnormal discharge from/burning or itching in vagina”
9. Healthy eating
10. How to apply algorithms

### Pre and post training assessment

In order to test the baseline knowledge of FHWs about reproductive health issues in adolescent girls a pre-training assessment was conducted. This was done using a piloted, structured, 30-item, self-administered questionnaire. It covered all the topics that were taught in the training. The 30 item questionnaire included multiple choice questions and case scenarios for testing the FHWs under three domains. These were knowledge (theoretical aspect of anatomy and physiology of female reproduction), evaluation (examining the problem and judging carefully), and application (decision making in the management of the problem). Details can be accessed in Additional file 1. All questions were scored after giving differing weightage to these domains. This meant questions which tested the application domain carried more marks compared to others. The maximum score was 38. The questionnaire took around 30 min to complete. Participants were encouraged to attempt all questions. Responses were kept anonymous to minimize non response rate. On the same day, after the completion of training, a post-training assessment was carried out. A repeat assessment was also carried out after 12 months, using the same questionnaire to the same FHWs. This was done to test the retention and usefulness of the training.

### Preparation and application of algorithms

The WHO Adolescent Job Aid has algorithms both for boys and girls including married adolescents. The present study was done only in unmarried girls aged 14–19 years (mid and late adolescence). Also, there was an issue of acceptance by young boys as the investigator was a female doctor. Therefore, conditions applicable only to unmarried girls were selected (Table 2). Job Aid algorithms were translated into Hindi and modified according to the local setting. According to the Job Aid, presenting complaints were assessed along with *ask, look, listen, feel* columns by the FHWs. Using the algorithm, the FHWs were guided to a particular classification of the problem. Classification of the condition is followed by relevant management and advice provided in the algorithm. Assessment of nutrition status was required in one of the algorithms. So, the color coded WHO body mass index (BMI) chart for 5–19 year old girls were given to the FHWs to identify malnutrition. Details can be accessed in Additional files 2 and 3.

**Table 2:** Algorithms included in the study

“Delayed puberty, female”
“I have lot of pain during my periods”
“I bleed a lot during my periods”
“I have irregular periods/my periods have stopped”
“I have abnormal discharge/burning/itching in vagina”.

### Field testing of algorithms

After training, field testing was carried out in which all the FHWs were asked to use the algorithms among adolescent girls in their respective sub-centers, to give them hands-on experience. Shortly after the hands-on exercise, a convenience sample of atleast ten girls per FHW, i.e., total number of 120 girls from all 12 subcentres were included for testing agreement. All the FHWs applied the algorithms independently. After the diagnosis and management according to the algorithm was done by the FHWs, the investigator independently applied the algorithms in all the girls, and the findings were compared later for agreement. In addition, a focus group discussion with all the FHWs was carried out at the end of the study to summarize their experiences while using the algorithms and their feedback was taken. This was moderated by a faculty member at CRHSP who had experience in conducting FGDs. This was conducted with the help of FGD guide which had open ended questions related to training and application of algorithms.

### Ethical issues

The protocol of the thesis was cleared by Ethics Committee of All India Institute of Medical Sciences. Informed consent and assent whenever applicable was obtained from the participants. Appropriate diagnosis and management according to the algorithm was followed.

### Statistical analysis

Data were entered in Epi Info 3.1.5 and the access file was transferred into a Stata file. All the analysis was carried out in Stata version 9. The mean scores were calculated for pre-training and post-training assessment of health workers. A non-parametric test was applied (Wilcoxon sign rank test) for the analysis of paired data. Results were expressed as mean, median and inter quartile range. Since we also wanted to know the agreement between the FHWs and the investigator, in diagnosing various conditions, Kappa was calculated with 95 % confidence intervals. Assuming the investigator’s diagnosis of each condition as gold standard in field conditions, the sensitivity, specificity, positive predictive value, and negative predictive value were also calculated.

## Results

### Results of pre and post training assessment

There were altogether 13 FHWs for whom pre and post training assessment was done. In the pre assessment the mean score of the participants was 19.9 (SD-4.5) while the median score was 19.2. The mean and median scores of post training assessment were 23.8 (SD-2.7) and 25.2 respectively and the difference was statistically significant (*p*–0.0071). Mean score of the participants 12 months after the training was 23.8 (SD-5.0), the median score was 23.2 and the scores ranged from 16.8 to 32.2. It was also found that compared with the pre- training scores, the difference was significant (*p*–0.0099) after one year of training (Table 3).

**Table 3:** Results of the pre and post training observation (*n* = 13)

Variables	Pre training	Immediately after training	One year after training
No of observations	13	13	13
Mean score	19.9 (SD 4.5)	23.8 (SD 2.7)	23.8 (SD 5.0)
Median score	19.2	25.2	23.2

### Agreement

Algorithms for five conditions were tested and assessed for agreement between the investigator and the FHWs. A total of 144 girls were assessed by the FHWs and the investigator (Table 4). One algorithm was applied on each adolescent girl depending on the chief complaint. According to the investigator’s classification the most common condition was dysmenorrhoea (48.6 %) followed by white discharge per vaginum (normal white discharge per vaginum) (17.4 %), reproductive tract infection (10.4 %) and menorrhagia (7.6 %). 13 girls were classified in the ‘normal’ category by the investigator; and of these, the health workers identified eight girls as ‘normal’. The maximum difference in classification by the FHWs and investigator were in the number of girls with normal white discharge per vaginum (more by investigator) and reproductive tract infection and menorrhagia (more by FHWs). Table 5 compares the classification of adolescent girls’ status as condition ‘present’ or ‘absent’ according to the assessment made by the FHWs and the investigator. The FHWs correctly classified 108 as true positives and 30 as true negatives. The overall agreement between the FHWs and the investigator was classified as good, with a kappa of 0.7 (95 % CI 0.5–0.9). In addition to the kappa statistic, we also calculated sensitivity and specificity and predictive values, assuming the investigator’s diagnosis as gold standard for field conditions for this tool. It was seen that the sensitivity, specificity, positive predictive value (PPV) and negative predictive value (NPV) were 94.3 %, 78.9 %, 92.5 % and 83.3 % respectively.

**Table 4:** Conditions assessed and classified by investigator and FHWs using algorithms (*n* = 144)

Conditions classified	Investigator	FHWs
Dysmenorrhoea	70	70
Menorrhagia	7	11
Menorrhagia with anaemia	4	4
Irregular periods of adolescence	6	8
Delayed puberty (normal)	3	3
Reproductive tract infection	19	15
White discharge per vaginum (normal)	22	25
Normal	13	8
Total	144	144

**Table 5:** Calculation of overall agreement for classifying the conditions according to the algorithm by health workers and investigator

	Investigator			Total	Validity parameters (95 % CI)
FHWs		Condition present	Condition absent	1	Sensitivity 94.3 % (88.2–97.4)
	Condition present	100	8	108	Specificity 78.9 % (63.6–88.9)
	Condition absent	6	30	36	Positive predictive value 92.5 % (86.0–96.2)
Total		106	38	144	Negative predictive value 83.3 % (68.1–92.1)
Kappa (95 % CI)	0.7 (0.5–0.9)				

### Results of FGD

All the FHWs participated in the FGD, and gave a positive feedback about training received and their ability to use the algorithms in the field. They found that algorithms were clear, simple leading to the subsequent steps in management of the problem. The difficulties reported were related to calculation of BMI and in convincing adolescent girls regarding issues like unnecessary use of analgesics for dysmenorrhoea, (Table 6).

**Table 6:** Results of Focussed group discussion

“When we can tell who is weak and who is well just by looking at them, then what is the need of BMI…moreover calculation is a bit difficult”
“Nowadays girls won’t do anything they don’t even go to school and they will start crying. If you give medicines they consider it as pleasance”

## Discussion

In the present study a consistent and significant difference between pre and post training knowledge scores of the FHWs was observed even after twelve months of training. Therefore we conclude that training was successful in increasing the knowledge of the FHWs about adolescent girls’ reproductive health issues. Increasing this knowledge was very crucial for enabling the FHWs to use algorithms. Another aspect is the duration of training which was one day. Since the FHWs included in the study were all ANMs which meant that they knew the basics of reproduction. So, according to us one day training was sufficient for them. Because of the training as well as hands on exercise, it was possible for the FHWs to use the modified Job Aid algorithms with ease. One of the reasons for misclassification of adolescent girls’ conditions (Table 4) might be poor adherence to algorithms by FHWs. For instance it has been shown that sensitivity for algorithm- based diagnosis by health workers was more in high-prevalent settings, even for diseases like tuberculosis [16]. Even though adolescent girls’ problems may not be as complicated as tuberculosis, still improper application of algorithms may lead to misdiagnosis. The conditions that are more prevalent like dysmenorrhoea are likely to have high sensitivity and positive predictive value. But, uncommon conditions like delayed puberty, could be correctly classified by strictly adhering to the algorithm. By using these modified algorithms in such conditions, it would be possible to help the FHWs to address common problems faced by adolescent girls. The predictive values were also high indicating that FHWs can be used for identification and classification of adolescent girls’ problems. In this study the FHWs reported that applying algorithms was not difficult, as these algorithms were modified for better user friendliness after pre testing.

Many studies done in developing countries have found that health providers as well as the adolescents perceive that the health services are not adolescent friendly [17–20]. During the course of study problems related to communication with adolescents was observed with few FHWs. The authors feel that in order to make adolescent friendly services, providers’ communication skills needs to be improved. Through our training FHWs were sensitised about communicating with adolescent girls and their guardians. We recommend that capacity building of health workers for improving communication skills should be part of any health programme dealing with adolescents.

A study done in Uganda among community reproductive health workers showed that they needed more training and wanted to include community members to deal with the myths and misconception in their communities [8]. For instance, self-medication needs to be discouraged, and there are studies which reported that self- medication for dysmenorrhoea is a common practice [21, 22]. In the present study when asked about the satisfaction of adolescents, most of the FHWs agreed that it was difficult to convince girls and their parents to avoid unnecessary self medication (use of analgesics). The authors believe that health workers must be sensitized about such issues. Health workers are to be trained so that they are in a better position to counsel adolescents and their elders.

The limitations of this study are the number of health workers trained is small. However, as this is a pilot study, we assume that inclusion of all the FHWs in the project area provided good information about the ease and usefulness of application of the algorithms in the field as a part of their routine work. Secondly, FHWs were trained in the application of the algorithm under ideal conditions without accounting for the field conditions which is normally might not be so conducive due to lack of time and privacy. Feasibility from the health workers’ perspective would have given more insight into practicality aspects. Thirdly, while calculating the agreement between the FHWs and the investigator, though the overall sample size for calculating kappa was adequate for all conditions together, the number of girls assessed for individual conditions separately was small; therefore agreement of each condition could not be assessed.

This study deals with the feasibility of involving the FHWs in addressing adolescent girls’ reproductive health problems using modified WHO algorithms for common conditions. It was seen that involving health workers was feasible as seen by good agreement between the assessment by the investigator and the lay health workers. But, other issues like time management during routine work, timing of training, and overhead cost of training etc., were not taken into account. Besides agreement, these issues would have given more insight into logistics part of feasibility of using these algorithms in providing adolescent health services at primary level.

## Conclusions

Adolescents need a safe and supportive environment where knowledge, counseling and treatment go hand in hand, which can be tailor made according to the local conditions. Through this study it was shown that with adequate training, female health workers can satisfactorily classify the common adolescent girls’ reproductive problems using the modified WHO Job Aid algorithms. There is therefore a need to focus on providing adolescent friendly services at primary level by trained health personnel in order to ensure that this generation of adolescents will in turn safeguard the health of future generation.

## Additional files

Additional file 1:
**Assessment questions used during pre and post training assessment.** (DOCX 16 kb) Additional file 2:
**Modifications done during preparation of algorithms.** (DOCX 72 kb) Additional file 3:
**Modified WHO Job Aid Algorithms.** (PDF 126 kb)
