# Supplementary material for: Feasibility and validity of using WHO adolescent job aid algorithms by health workers for reproductive morbidities among adolescent girls in rural North India
Source: BMC Health Serv Res. 2015 Sep 21;15:400. doi: 10.1186/s12913-015-1067-x (PMC4578239; doi:10.1186/s12913-015-1067-x)
Supplement: Additional file 1: — Assessment questions used during pre and post training assessment. (DOCX 16 kb) [file 12913_2015_1067_MOESM1_ESM.docx]

**Assessment questions**

**Additional file: 1 Assessment questions used during pre and post training assessment**

Form no-

Date:

1. What is the normal duration of a menstrual cycle?
   1. 10-15 days
   2. 21-45 days
   3. 50-100 days
2. What do you mean by fertilization?
   1. Separation and shedding of inner layer of uterus
   2. Shedding of egg from the ovary
   3. Both
   4. None of the above
3. According to the marked numbers identify the female reproductive organs


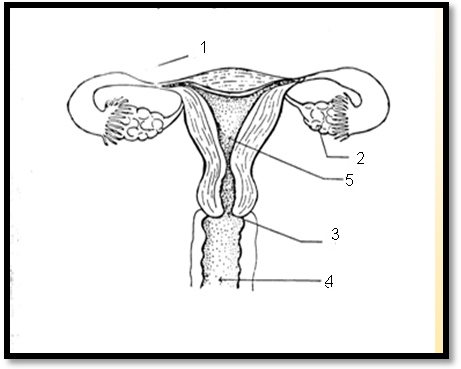


1. 2. 3. 4. 5.

1. Chose the best answer among the two

The deciding factor in determining the sex of the baby will be

- 1. Mother
  2. Father

1. What is the main cause for painful periods during adolescence?
   1. Hormones responsible for menstruation
   2. Chemicals released during menstruation
   3. Due to consumption of certain food stuffs
   4. Due to some disease conditions
2. What do you understand by delayed puberty?
   1. If there is no breast development by age 14 years
   2. Not menstruating by age 16 years
   3. Not menstruating by age 20 years
   4. Both a and or b
   5. Both a and or d
3. When is the pain maximum during menstruation?
   1. During 3^rd^ or 4^th^ day of menstruation
   2. 2 days before and 2 days after starting of menstruation
   3. Pain will be same on all the days of menstruation
   4. None of the above
4. What is the usual time when girls enter puberty?
   1. 5-10 years
   2. 9-14 years
   3. 16-20 years
5. A mother is worried that her 12 years old daughter has not developed signs suggestive of puberty. As a health worker what will you do in this case?
   1. Refer her for doctor consultation at primary health centre
   2. Calculate body mass index of her daughter
   3. Reassure mother
   4. All of the above
6. During adolescence what is the most common reason for excessive bleeding during menstruation?
   1. Body is still developing and is not fully mature yet
   2. Bleeding disorders
   3. Any long standing illness
   4. Under nutrition
7. Say yes or no

“During adolescent stage girls do not require extra nutritious diet”

1. What do you understand by safe period?
   1. Menstrual period without any problems
   2. A method of contraception
2. Say yes or no

“In an adolescent girl menstrual period would stop only when she is pregnant”

1. During adolescence irregular menstrual periods is very common
   1. Agree
   2. Disagree
   3. Don’t know
2. Himanshi is a 16 years old girl having itching in external genitalia and white discharge per vaginum. During menstruation since she has to take rest she is not even allowed to take bath.

Say if this is right or wrong giving relevant reasons.

1. Say yes or no

“In an adolescent girl one of the reasons for irregular menstrual periods is stress such as sleep deprivation etc.”

1. In those adolescent girls having irregular menstrual periods there is always a need to investigate for irregular periods.
   1. Agree
   2. Disagree
   3. Don’t know
2. Say if the following management if right or wrong giving relevant reasons.

Radha is suffering from ulcer in her genitalia and also her husband is also suffering from similar complaints. ASHA worker adviced Radha to go to sub centre for treatment even though her husband could not go with her.

1. Sheela is a very shy girl and she studies in 9^th^ standard. She came to the subcentre along with her mother with complaints of white discharge per vaginum. As a health worker how will you manage her problem?
   1. Talk to Sheela in private before giving any advice
   2. Ask few questions to her mother before any advice
   3. Tell her about her condition in detail before any advice
   4. Without asking anything refer her to primary health centre
2. What all changes will happen when a girl attains puberty?
   1. Increase in height and change in voice
   2. Development of hair near axilla, genitalia
   3. Occurrence of acne
   4. Increase in the size of breasts
3. Vimla is a 17 year old married girl who came to meet Kamini, health worker suspecting that she might be pregnant.

*Kamini*: When did you last get your periods?

*Vimla*: I don’t remember exactly. Last time I had periods when I went to *basant mela,* which was 2 months back.

*Kamini*: Cant you even remember such minor things and you expect us to help you. I think you might be 3 months pregnant.

*Vimla*: Yes it could be possible. You are right!

*Kamini*: You have to come on Monday for check up

*Vimla*: Okay (in a disappointed tone). I have to ask you something...

*Kamini*: (interrupting *Vimla*) come on Monday and ask everything on Monday itself i don’t have time now.

Do you think about this conversation between health worker and a married adolescent girl is effective?

If yes then justify with reasons.

If no then how this conversation can be improved to make it effective?

1. Mark all the symptoms of reproductive tract infections among adolescent girls
   1. White discharge per vaginum
   2. Back pain
   3. Pain abdomen
   4. Itching genitalia
   5. Burning micturition
   6. Ulcer over external genitalia
2. What causes menstrual periods?
   1. Excessive bodily heat
   2. Supernatural powers due to which bad blood is removed from the body
   3. Physiological process due to hormones
   4. All of the above
3. Which nutrient is present in large amount in green leafy vegetables?
   1. Calories
   2. Calcium
   3. Vitamins
   4. Iron
   5. All of the above
4. Mark all the symptoms of anaemia
   1. Loss of appetite
   2. Irregular menstrual periods
   3. Palmar pallor
   4. Lethargy
5. What options are available for managing painful periods during adolescence?
   1. Medications
   2. Continue ones work or studies
   3. Consumption of hot tea
   4. Oral contraceptive pills
   5. Maintenance of personal hygiene during periods
6. Which food items needs to be consumed more in anaemia?
   1. Rice
   2. Ground nuts
   3. Green leafy vegetables
   4. Banana
7. Mark all those food items which is having more calcium.
   1. Curd
   2. Eggs
   3. Pulses
   4. None of the above
8. What are the ill effects of eating junk foods?
   1. Infections
   2. Empty calories
   3. Malnutrition
   4. None of the above
9. Say yes or no

“Consuming foods that are nutrient deficient could lead to anaemia in adolescent girls”
