# Supplementary material for: Feasibility and validity of using WHO adolescent job aid algorithms by health workers for reproductive morbidities among adolescent girls in rural North India
Source: BMC Health Serv Res. 2015 Sep 21;15:400. doi: 10.1186/s12913-015-1067-x (PMC4578239; doi:10.1186/s12913-015-1067-x)
Supplement: Additional file 2: — Modifications done during preparation of algorithms. (DOCX 72 kb) [file 12913_2015_1067_MOESM2_ESM.docx]

**MODIFICATION DONE IN THE PREPARATION OF ALGORITHMS**

**Additional file: 2 Modifications done during preparation of algorithms**

In all the algorithms these have been skipped

- Sexual reproductive health screen
- HEEADSSS assessment signs
- Symptoms of STI

In every algorithm to assess if the girl is sexually active by asking whether she is married or not

| **Sl no** | **WHO Adolescent Job Aid** | **Modified** | **Reason** |
| --- | --- | --- | --- |
| 1 | **Delayed puberty**  In *ASK*   - Do a sexual reproductive health screen - DO HEEADSSS assessment   In *MANAGE & FOLLOW UP*   - Manage and follow up in separate column | - Both have been skipped - Management and follow up have been combined in a single column - Algorithms like “I am too fat /thin” has been skipped | - As the girls interviewed would not have attained puberty it would be too early to ask this in our set up and also does not contribute towards the classification of the condition of girls - Second one would be time consuming and for that separate assessment material needs to be prepared ^*^ - To make one algorithm in a single page management and follow up have been combined - These algorithms are not given in WHO guide |
| 2 | I have pain during my periods  In *LOOK FEEL LISTEN*   - Presence of abdominal mass - Palpable uterus in lower abdomen   In *Manage* | - Both have been skipped - Abdominal examination repeated with no signs of pregnancy - Dosage according to weight has been removed | - Very rare finding to be commonly find out - Uterus will be palpable inside the pelvis in the first 3 months - In order to avoid confusion only one dosage has been given |
| 3 | I bleed a lot during my periods  In *ASK*   - Do you use DMPA injections   In *LOOK FEEL LISTEN*   - Enlarged uterus on vaginal examination - General physical examination   In *MANAGE*   - Menorrhagia associated with DMPA | - DMPA injections has been removed - Vaginal examination has been skipped - Limited to only checking for signs of anaemia - Menorrhagia related to DMPA has been removed | - Use of DMPA injections is very uncommon - Vaginal examination is not feasible - As only signs of anaemia only is addressed in the management - Menorrhagia associated with DMPA is very rare given its use is uncommon |
| 4 | **I have irregular periods**  In *LOOK FEEL LISTEN*  Vaginal examination | - Vaginal examination has been removed - DMPA has been removed | - Vaginal examination is not feasible - Use of DMPA injections is very uncommon |
