# Supplementary material for: Feasibility and validity of using WHO adolescent job aid algorithms by health workers for reproductive morbidities among adolescent girls in rural North India
Source: BMC Health Serv Res. 2015 Sep 21;15:400. doi: 10.1186/s12913-015-1067-x (PMC4578239; doi:10.1186/s12913-015-1067-x)
Supplement: Additional file 3: — Modified WHO Job Aid Algorithms. (PDF 126 kb) [file 12913_2015_1067_MOESM3_ESM.pdf]

Additional file 3: Modified WHO Job Aid Algorithms

मुझे मासिक धर्म के समय बहुत दर्द होता है ः

पूछें	देखें/महसूस/करें सुनें	लक्षण	वर्गीकरण	प्रबन्धन
**दर्द**<br>• क्या आपको पहले भी ऐसा दर्द हुआ है<br>• क्या यह दर्द सामान्यतः आपको मासिक धर्म के समय हुआ, या मासिक धर्म के बीच में<br>• क्या आपको अभी रक्तस्राव हो रहा है / क्या आपको अभी मासिक धर्म हो रहा है<br>• क्या अभी आपको दर्द हो रहा है<br>• मासिक धर्म जैसा है / या यह बहुत कम / बहुत ज्यादा है<br><br>**अगर शादी हुई हैं तों** (यौन रूप से सकिय हैं)	**पेट की जॉंच**<br>• निचले पेट में दर्द हैं<br>• क्या दर्द हल्का/थोड़ा ज्यादा/ बहुत ज्यादा है<br><br>**गर्भावस्था** (यौन रूप से सकिय हैं)<br>अगर यौन रूप से सकिय है तो ः<br>– गर्भनिरोध का प्रयोग सही ढंग से और लगातार नहीं करते<br>– या उसे मासिकधर्म देर से होता है<br>– या उसे गर्भावस्था के कोई लक्षण है<br><br>**गर्भावस्था के लक्षण देखें**<br>• गर्भावस्था जॉंच करें	अभी दर्द हैं<br>और<br>गर्भावस्था हो सकती हैं<br>• अगर यौन रूप से सकिय है तो<br>• अगर गर्भनिरोधक प्रयोग नहीं करती है तो<br>• मासिक देर से आया हैं और गर्भावस्ता का कोई लक्षण हैं<br>और<br>• अभी रक्तस्राव हो रहा है और रक्तस्राव सामान्य मासिक धर्म जैसा नहीं हैं<br>• दवाने से पेट मे दर्द होता हैं	**Possible threatened abortion/ectopic pregnancy**<br>**संभाविक गर्भपात / अस्थानिक गर्भावस्था**	Urgent referral to Medical Officer/Gynaecologist(स्त्री रोग विशेषज्ञ)
• क्या आप गर्भ की रोकथाम के लिए कोई गर्भ निरोधक प्रयोग करती है<br>• आप कौन सा तरीका अपनाती है<br>• अगर मौखिक गर्भनिरोधक गोली का प्रयोग करती है तो ः क्या आप कभी अपनी गोली लेना भुली है<br>• अगर कन्डोम प्रयोग करती है तो ः क्या आप कन्डोम हर समय प्रयोग करती है जब भी सम्भोग करती है	**पेट की जॉंच करें**<br>• निचले पेट में दर्द हैं<br>• क्या दर्द हल्का/थोड़ा ज्यादा/ बहुत ज्यादा है<br><br>**और**<br><br>**अगर शादी हुई हैं तों** (यौन रूप से सकिय हैं)<br><br>**गर्भावस्था का कोई लक्षण नहीं हैं**	• अगर मासिक धर्म के पहले और बीच मे दर्द होता हैं<br>• अगर अभी दर्द हैं<br>• दवाने से पेट मे दर्द नहीं होता हैं<br>• अगर अभी रक्तस्राव हो रहा हैं<br>• रक्तस्राव सामान्य मासिक धर्म जैसा हैं<br><br>और<br><br>• गर्भावस्ता नहीं हो सकती हैं	**Dysmenorrhoea**	Treat pain:<br>Hot fomentation when she gets pain<br><br>If this does not control pain, then it requires medications so refer to Medical Officer for initiation of treatment.<br><br>Advise to take tablets as soon as pain begins<br>Do not give for more than 7 days<br><br>Advice her to continue with her daily activities<br><br>Follow up:<br>Follow up after 3 months<br><br>If there is no improvement after 3 months then refer to Medical Officer

मुझे मासिकधर्म के दौरान बहुत रक्तस्राव होता है ः

पूछें	देखें/महसूस/करें सुनें	लक्षण	वर्गीकरण	प्रभन्दन
**मासिक धर्म के समय**<br>• क्या आपको अभी मासिकधर्म/रक्तस्राव हो रहा है<br>• आपको मासिकधर्म कितने दिनों में वंद हो जाता है<br>• मासिकधर्म के समय कितने सेनिटेरी नैपकिन/पेड/ रूई के पैड/ अन्य उपकरण प्रयोग हो जाते है<br><br>**अगर शादी हुई हैं तो** (यौन रूप से सक्रिय हैं)<br>• क्या आप गर्भ की रोकथाम के लिए कोई गर्भनिरोधक प्रयोग करती है<br>• अगर हॉ तो आप कौन सा तरीका प्रयोग करती है<br>• अगर कापर टी का प्रयोग नहीं करती है ः<br>• अगर कन्डोम प्रयोग करती है तो ः क्या आप हर समय जव भी संभोग करती है कंडोम का प्रयोग करती है<br>• अगर मौखिक गर्भनिरोधक गोली का प्रयोग करती है तो ः क्या आप कभी अपनी गोली लेना भूली है<br><br>**गर्भावस्था**<br>• क्या आपका एक मासिकधर्म छूट गया या आपको मासिक धर्म देर से हुआ<br>• क्या आपको निम्न में से कोई लक्षण है ः<br>– सुवह मिचली / उल्टी<br>– स्तन में भारीपन या दर्द<br><br>**खून की कमी (अनीमिया)**<br>• क्या आप हर समय थका हुआ महसूस करती है	**गर्भावस्था**<br>अगर यौन रूप से सक्रिय है तो ः<br>– गर्भनिरोधक का प्रयोग सही ढंग से और लगातार नहीं करते<br>– या उसको मासिकधर्म देर से होता है<br>– या उसको गर्भावस्था के कोई लक्षण है<br>**देखें**<br>नीचली पेट में बच्चेदानी को<br>**करे**<br>गर्भावस्था (पेशाव) की टेस्ट	अगर अभी मासिकधर्म/रक्तस्राव हो रहा है<br>और<br>गर्भावस्था<br>और<br>गर्भावस्था होने की संभावना हैं<br>• यौन रूप से सक्रिय है<br>– गर्भनिरोध का प्रयोग सही ढंग से और लगातार नहीं करते<br>– या उसको मासिकधर्म देर से होता है<br>– गर्भावस्था का कोई लक्षण हैं	**Possible threatened abortion/ectopic pregnancy**<br>**संभाविक गर्भपात / अस्थानिक गर्भावस्था**	Urgent referral to Medical Officer/Gynaecologist(स्त्री रोग विशेषज्ञ)
	**मासिकधर्म के दौरान अत्यधिक रक्तस्राव होता हैं**<br>**और**<br>**गर्भावस्था का काई लक्षण नहीं हैं**<br>और<br>**खून की कमी (अनीमिया) का कोई लक्षण हैं**<br>जॉच के लिए<br>• हथेली का फीकापन<br>• ऑख की निचली झिल्ली का फीकापन<br><br>हीमोग्लोविन टेस्ट करें	• अगर **7** से ज्यादा पेड को वदलना पडता है<br>• **7** दिन से ज्यादा रक्तस्राव हो रहा है<br>• अनीमिया कि कोई लक्षण<br>–हथेली का फीकापन<br>–ऑख की निचली झिल्ली का फीकापन<br>• हीमोग्लोविन **12** से कम है	**Menorrhagia with anemia**	For regulating bleeding<br>Needs treatment so refer to Medical Officer for initiation of treatment<br><br>Treat anaemia<br>Iron –Folic acid tablets 200mg<br>Start 1 tablet orally 3 times per day<br><br>Treat for 3 months<br>Follow up for drug supply after 3 months
	**मासिकधर्म के दौरान अत्यधिक रक्तस्राव होता हैं**<br>**और**<br>**खून की कमी (अनीमिया) का कोई लक्षण नहीं हैं**	• अगर **7** से ज्यादा पेड को वदलना पडता है<br>• **7** दिन से ज्यादा रक्तस्राव हो रहा है<br>• अनीमिया कि कोई लक्षण नहीं हैं<br>• हीमोग्लोविन **12** से ऊपर हैं<br>• कांपर टीं का इस्तेमाल नहीं कर रही हैं	**Menorrhagia with no anemia**	Regulate bleeding (as above)<br>*AND*<br>Prevent anaemia<br>Iron –Folic acid tablets 200mg<br>1 tablet orally 1 time per day<br><br>Treat for 3 months<br>Review after 3 cycles
	**मासिकधर्म के दौरान अत्यधिक रक्तस्राव होता हैं**<br>**और**<br>**गर्भावस्था का काई लक्षण नहीं हैं**<br>**और**<br>अगर कांपर टी का प्रयोग करती हैं तो	• अगर **7** से ज्यादा वार पेड को वदलना पडता है<br>• **7** दिन से ज्यादा रक्तस्राव हो रहा है<br>और<br>• कांपर टीं का इस्तेमाल कर रही हैं	**Menorrhagia possibly associated with contraception method (Copper T)**	Regulate bleeding (as above)<br>Treat/ Prevent anaemia (as above)<br><br>After 3 months if heavy bleeding continues discuss the removal of Copper T and starting alternative methods

मुझे अनियमित मासिकधर्म होता है मेरा मासिकधर्म बंद हो गया है

पूछें	देखें/महसूस/करें सुनें	लक्षण	वर्गीकरण	प्रभन्दन
**अनियमित मासिकधर्म**<br>• आप कितने साल की थी जब आपको मासिकधर्म शुरू हुआ<br>• क्या आपका मासिकधर्म सामान्यत:नियमित है (क्या यह हर महीने उसी समय पर आता है)<br>• अगर महीना अनियमित हैं तो यह कब से अनियमित हैं<br>• यह कितने दिन तक होता है<br>• क्या आपको मासिकधर्म के बीच में दाग या रक्तस्राव होता है? क्या यह अकसर आता है?<br><br>**अगर शादी हुई हैं तो** (यौन रूप से सक्रिय हैं)<br>• क्या अगर आप पिचले 6 महीने में या अभी कोई गर्भनिरोधक का इस्तेमाल कर रहा है<br>• यदि अभी इस्तेमाल कर रहे हो ः कौन सा तरीका इस्तेमाल कर रहे हो<br>• अगर गर्भनिरोध के लिए कन्डोम प्रयोग करती है तो ः क्या आप हर समय जब भी संभोग करती है कंडोम का प्रयोग करती है<br>• अगर मौखिक गर्भनिरोधक गोली का प्रयोग करती है तो ःक्या आप कभी अपनी गोली लेना भूली है<br>**गर्भावस्था**<br>• क्या आप यह सोचती है कि आप गर्भवती हो सकती है<br>• क्या आपका एक मासिकधर्म छूट गया या आपको मासिक धर्म देर से हुआ<br>• क्या आपको निम्न में से कोई लक्षण है ः<br>– सुबह मिचली / उल्टी<br>– स्तन में सूजन या दर्द	**अगर यौन रूप से सक्रिय है तो ः**<br>– गर्भनिरोध का प्रयोग सही ढंग से और लगातार नहीं करते हैं<br>– या उसको मासिकधर्म देर से होता है<br>– या उसको गर्भावस्था के कोई लक्षण है<br>**देखें**<br>नीचली पेट में बच्चेदानी को<br><br>**करे**<br>गर्भावस्था (पेशाव) की टेस्ट	अगर अभी मासिकधर्म/रक्तस्राव हो रहा है<br>और<br>गर्भावस्था<br>और<br>गर्भावस्था होने की संभावना हैं<br>• यौन रूप से सक्रिय है<br>– गर्भनिरोध का प्रयोग सही ढंग से और लगातार नहीं करते<br>– या उसको मासिकधर्म देर से होता है<br>– गर्भावस्था का कोई लक्षण हैं	**Possible threatened abortion**<br>***OR***<br>**Possible ectopic pregnancy**<br>**संभाविक गर्भपात / अस्थानिक गर्भावस्था**	Urgent referral to Medical Officer/Gynaecologist(स्त्री रोग विशेषज्ञ)
		अगर पहला मासिकधर्म 2 साल पहले हुआ था आनियमित मासिकधर्म<br>• पिचले 3 महीनों से कोई मासिक धर्म नहीं आया<br>• मासिकधर्म का चक्र 21 दिन से कम है या 35 दिन से ज्यादा हैं<br>• दोनों मासिकधर्म के बीच का अन्तराल 20 दिन से ज्यादा बदलता रहता हैं<br>• मासिकधर्म के बीच में दाग या रक्तस्राव होता है<br>और<br>हारमोनल गर्भनिरोध का इस्तेमाल नहीं करती हैं	**Irregular periods Not associated with hormonal contraceptives**	Refer to Medical Officer/ Gynaecologist(स्त्री रोग विशेषज्ञ)
		अनियमित मासिकधर्म (ऊपर बताया गया)<br>और<br><br>पिचले 6 महीने में या अभी हारमोनल गर्भनिरोध का इस्तेमाल कर रही हैं<br>• माला एन्	**Irregular periods Associated with hormonal contraceptives**	If using OCPs* and not taking it regularly Advice her to use alternative methods like condom until she has been taking OCPs regularly for atleast 7 days<br><br>If taking OCPs regularly<br>If this is less than 4 months advice her that irregular bleeding is common during this period<br><br>If taking OCPs for more than 4 months then refer
		अगर पहला मासिकधर्म 2 साल मे हुआ था अनियमित मासिकधर्म जैसे (ऊपर बताया गया)	**Menstrual irregularity of early adolescents**	Reassure that irregular bleeding is common in first 2 years and periods are likely to become regular with time.

*OCP- Oral contraceptive pills like Mala N, Mala D

यौवन अवस्था में देरी

<table>
<tr><th>पूछें</th><th>देखें/महसूस/करें सुनें</th><th>लक्षण</th><th>वर्गीकरण</th><th>प्रभन्दन</th></tr>
<tr>
<td rowspan="3">• आप कितने वर्ष की है?

स्तन (वक्ष) विकास
• क्या आपने अपने स्तन के आकार में कोई परिवर्तन देखा है?
अगर स्तन विकास शुरू हुआ तो
• आप कितने वर्ष की थी जब आपने यह परिवर्तन देखा था?

Pubic hair* (जघन बाल)
• क्या आपके शरीर में जननांगो के पास बाल उगे?
अगर जघन बाल है तो ः
• आप कितने साल की थी जब आपने यह खयाल किया?

मासिक धर्म (महिना)
• क्या आपको मासिक धर्म (महिना) शुरू हो गया है?
अगर मासिक धर्म (महिना) शुरू हो गया है तो ः
• आप कितने साल की थी जब आपको पहला मासिक धर्म शुरू हुआ

दीर्घस्थायी बीमारी
• क्या आपको कोई पुरानी (लंबे समय से) बीमारी है
(नोट: अगर लंबे समय से चल रही बीमारी के कोई लक्षण जैसे ः बुखार, खांसी, दस्त, वजन कम होना इत्यादि)</td>
<td rowspan="3">जॉच करना
• वजन
• लंबाई

गणना करना
• BMI# = वजन / लंबाई X लंबाई

जॉच
• स्तन = स्तन tissue की उपस्थिति एवम् चूचुक (nipple) के आस पास का रंग और आकार
• Presence of pubic hair

जांच करें
• आगर लम्बे समय से बुखार ,खांसी ,दस्त, वजन का घटना इत्यादि हैं
–हथेली का फीकापन
–ऑख की निचली झिल्ली का फीकापन
–बुखार</td>
<td>• 14 वर्ष की उम्र तक स्तन का विकास न होना।
• 14 वर्ष की उम्र तक Pubic hair
• 16 वर्ष की उम्र तक महिना न होना।
• 5 साल से ज्यादा समय बीत गया जब स्तन विकास का पहला लक्षण दिखा परन्तु अभी तक पहला महिना नहीं आया।
और
• पोषण की कमी
• दीर्घस्थायी बीमारी के लक्षण</td>
<td>यौवन अवस्था में देरी

Possibly due to chronic illness
OR
Undernutrition</td>
<td>• Refer to Medical officer

• Address nutritional problems

• Review after 6 months for assessment of pubertal development</td>
</tr>
<tr>
<td>• 14 वर्ष की उम्र तक स्तन का विकास न होना।
• 14 वर्ष की उम्र तक Pubic hair
• 16 वर्ष की उम्र तक महीना न होना।
• 5 साल से ज्यादा समय बीत गया जब स्तन विकास का पहला लक्षण दिखा परन्तु अभी तक पहला महीना नहीं आया।
और
• पोषण की कमी और पुरानी बीमारी के लक्षण नहीं हैं</td>
<td>यौवन अवस्था में देरी

Unlikely to be due to chronic illness
OR
Undernutrition</td>
<td>• Advice her that pubertal development is delayed for her age

• Refer to Medical Officer

• Reassure that even though puberty is delayed, most girls will eventually go through puberty, Stress that a small number do not and that is why she needs to be checked further</td>
</tr>
<tr>
<td>• 13 साल की है या छोटी है
• अगर 14 साल या उस से बडी है तो स्तन विकास शुरू हो गया।
• अगर 14 साल या उस से बडी है तो Pubic hair है।
• अगर 16 साल या उस से बडी है तो महिना शुरू हो गया।
और
• 5 साल से कम समय बीत गया जब स्तन विकास का पहला लक्षण दिखा और उससे पहला महीना आया ।</td>
<td>Normal</td>
<td>• Reassure that even if the signs of pubertal development have not appeared, she is within normal limits for age

• Reassure that most girls will eventually develop and go through puberty</td>
</tr>
</table>

* Pubic hair: बाहरीय जननांग के पास बाल होना

#BMI: Body mass index. प्रशिक्षण पुस्तिका के अनुवन्ध को देखिये

मुझे मेरी जननेन्द्रिय (गुप्तांग/योनि) से असाधारण ज्वलनशील अथवा खुजली वाला स्राव होता हैं

<table>
<tr><th>पूछें</th><th>देखें/महसूस/करें सुनें</th><th>लक्षण</th><th>वर्गीकरण</th><th>प्रभन्दन</th></tr>
<tr>
<td rowspan="3">योनि से स्राव
क्या आप अपने स्राव की प्रकार के वारें में वताएंगी ?
• रंग ः क्या स्राव/साफ सफेद/हरा/ भूरा/पीला था
• स्थिरता ः क्या स्राव पतला या गाढा था
• दुर्गंध ः क्या स्राव में दुर्गंध थी
• खुजली या जलन ः क्या आपको जननेन्द्रियों (गुप्तांग/योनि) में खुजली या जलन होती है।
• निचले पेट में दर्द ः क्या आपको निचले पेट में दर्द होता हैं
– अगर पेट में दर्द हैं तो देखे
– हाल ही में प्रसव
– असामान्य योनि स्राव

अगर शादी हुई हैं तों (यौन रूप से सक्रिय हैं)
क्या आपको कोई और जननेन्द्रिय संवंधी समस्या है
• क्या आपको पेट और जॉंघ के वीच के भाग में सुजन है
• पति को कोई जननेन्द्रिय संवंधी समस्या है
• मूत्रत्याग में दर्द होना (पुरूष)</td>
<td rowspan="3">पेट की जॉंच
• निचले पेट में दर्द हैं
• क्या दर्द हल्का/थोड़ा ज्यादा/ वहुत ज्यादा है

सामान्य शरीरिक जांच (पहले उल्लेख किया गया)</td>
<td>असामान्य योनि स्राव है ः
• रंग ः अगर स्राव सफेद/हरा/ भूरा/पीला है
• स्थिरता ः अगर स्राव गाढा है
• दुर्गंध ः अगर स्राव में दुर्गंध है
• खुजली या जलन ः अग्र जननेन्द्रियों (गुप्तांग/योनि) में खुजली या जलन है
और
• निचले पेट में दर्द नहीं हैं</td>
<td>Reproductive tract infections (RTIs)</td>
<td>Refer to Medical Officer

Advice about completion of course of medications

Stress the importance of genital and menstrual hygiene</td>
</tr>
<tr>
<td>असामान्य योनि स्राव है ः
• रंग ः अगर स्राव सफेद/हरा/ भूरा/पीला है
• स्थिरता ः अगर स्राव गाढा है
• दुर्गंध ः अगर स्राव में दुर्गंध है
• खुजली या जलन ः अगर जननेन्द्रियों (गुप्तांग/योनि) में खुजली या जलन है
और
• निचले पेट में दर्द नहीं हैं
और
गोनोरिया (Gonorrhoea) या क्लामैडिया (Chlamydia) या कोई STI* होने कि आशंका है</td>
<td>Pelvic inflammatory disease(PID)

OR

Sexually transmitted disease(STI)</td>
<td>Refer to Medical Officer

Advice about completion of course of medications

Stress the importance of genital and menstrual hygiene

In married couples encourage consistent use of condoms(Nirodh)

Follow up after 1 week</td>
</tr>
<tr>
<td>सामान्य योनि स्राव है ः
• स्पष्ट है
• पतला है
• कोई वदवू नहीं है
• माहवारी चक्र के वीच में स्राव को थोड़ा गाढा या चिपचिपा होती है</td>
<td>Normal</td>
<td>Reassure that this is normal vaginal discharge

Advice the girl about maintaining genital hygiene</td>
</tr>
</table>

*STI- Sexually transmitted infections (यौन संक्रमण).

PID- Infection of Uterus, Fallopian tube, Ovaries etc. Most common cause is STI.
